# Proinflammatory gene polymorphisms are potentially associated with Korean non-Sjogren dry eye patients

**Published:** 2011-10-29

**Authors:** Kyung-Sun Na, Jee-Won Mok, Ja Yeon Kim, Choun-Ki Joo

**Affiliations:** 1 Department of Health Promotion Center, Seoul St. Mary’s Hospital, College of Medicine, The Catholic University of Korea, Seoul, Korea; 2 Catholic Institute for Visual Science, Seoul, Korea; 3 Department of Ophthalmology and Visual Science, Seoul St. Mary’s Hospital, College of Medicine, The Catholic University of Korea, Seoul, Korea

## Abstract

**Purpose:**

To determine whether proinflammatory cytokine genes were potential susceptibility candidate genes for Korean patients with non-Sjogren dry eye, we investigated the association of the interleukin 1 beta (*IL1B*), interleukin 6 (*IL6*), and interleukin 6 receptor (*IL6R*) variations with this disease in Korean patients.

**Methods:**

Genomic DNA was extracted from blood samples of unrelated non-Sjogren dry eye patients and healthy control individuals who visited the Eye Center and Health Promotion Center of St. Mary’s Hospital in Seoul, Korea. For screening genetic variations in proinflammatory cytokine genes, the 511 (rs16944) and 31 (rs1143627) positions in the promoter region of *IL1B*, rs1143634 in exon 5 of *IL1B*, rs1800795 of the *IL6* promoter, and Asp358Ala (rs8192284) of *IL6R* were genotyped using the polymerase chain reaction, restriction fragment length polymorphisms, and direct sequencing.

**Results:**

Among the polymorphisms, rs1143634 (F105F) in exon 5 of *IL1B* was significantly different between the patient and control groups. The frequency of the C/T genotype in dry eye patients was decreased relative to that of the control subjects (10.4% versus 3.9%, p=0.043, OR=3.337). For the *IL6R* gene, the genotypic and allelic distribution of rs8192284 was different between the dry eye patients and the controls: CC genotype (p=0.017, OR=2.12) and C allele (OR=1.26).

**Conclusions:**

This is the first report of genetic variation screening of proinflammatory cytokine genes in Korean non-Sjogren dry eye patients. It is suggested that rs1143634 of *IL1B* and rs8192284 of *IL6R* act as susceptibility variations in Korean non-Sjogren dry eye patients.

## Introduction

As defined by the International Dry Eye Workshop, dry eye disease (DED) is a multifactorial disease of the tears and ocular surface and results in symptoms of discomfort, visual disturbance, and tear film instability, with potential damage to the ocular surface. Two major etiological causes of DED is aqueous tear-deficiency dry eye (ADDE) and evaporative dry eye (EDE). And ADDE has two major subclasses, Sjogen dry eye and non-Sjogren dry eye. non-Sjogren dry eye is a form of ADDE due to lacrimal dysfunction, when the systemic autoimmune features have been excluded [1].

DED has been estimated to afflict 11%–22% of the general population, depending on the parameters and population studied [2]. Older women and urbanites seem to be more affected than the rest of the population [3,4]. Furthermore, systemic diseases such as arthritis and allergies are correlated with an increased incidence of DED [4]. Numerous conditions, including medications, hormonal changes, environment, and neural alterations are known to cause non-Sjogren DED [5]. Recent studies have focused on the use of accurate biologic markers and diagnostic applications in establishing treatment strategies to cover the multifactorial nature of non-Sjogren DED.

Although the exact mechanism of non-Sjogren DED pathogenesis is not fully understood, hyperosmolarity and the resultant inflammation are known to be key components [6]. Increased levels of proinflammatory cytokines and markers have been observed in the tears and the ocular surface of non-Sjogren DED patients. Upregulation of inflammatory cytokines, including interleukin-1α(IL1α and IL1β has been observed in non-Sjogren DED [7,8]. In addition, various experimental animal models have increased levels of tumor necrosis factor-α (TNF-α), matrix metalloproteinase-9 (MMP-9), interleukin 6 (IL6), IL1α and IL1β [9-11]. The ocular surface inflammation in non-Sjogren DED is sustained by an ongoing activation and infiltration of pathogenic immune cells, primarily of the cluster of differentiation 4 (CD4^+^) T cell compartment [12-15]. The contribution of T cells to the immunopathogenesis of non-Sjogren DED is supported by studies that have shown increased T cell infiltration of the conjunctiva in humans and animals [16-19]. The author questioned whether the inflammation of non-Sjogren DED is the cause or the consequences of the pathogenesis and whether the genetic susceptibility have some role in the occurrence of non-Sjogren DED.

In general, an immune-mediated inflammatory disease (IMID) represents a group of various chronic conditions that share a common pathway and is characterized by an unidentified etiology. However, both genetic and environmental factors play an important role in its pathogenesis [20]. Recently, genome-wide association (GWA) studies and genome-wide, non-synonymous single nucleotide polymorphism (nsSNP) scans in humans have led to the identification of several loci associated with disease susceptibility. Each population holds a specific mutational pool [21]. For these pools, most mutations have mild effects individually but in combination with other alleles, may promote or inhibit occurrence of an IMID [22].

To determine whether polymorphisms altering the function or expression of proinflammatory cytokine genes contribute to the pathogenesis of non-Sjogren DED, we screened the proinflammatory cytokine genes interleukin 1 beta (*IL1B*), *IL6*, and the IL6 receptor (*IL6R*) gene. To the best of our knowledge, this study represents the first report of genetic variation screening for proinflammatory cytokine genes associated with non-Sjogren DED.

## Methods

### Study subjects and blood samples

The study sample included 251 patients with non-Sjoren DED and 109 healthy controls. Written informed consent was obtained from all subjects, and study was approved by the Medical Ethics Committee of the Catholic University of Korea, Seoul, Korea. Blood samples were collected after enrollment and were centrifuged at 405× g for 15 min to obtain plasma that was then aliquoted and stored at −70 °C until the day of analysis.

### Data on DED

Eligible patients were at least 21 years of age and had been diagnosed with DED. The inclusion criteria were as follows: Schirmer test (without anesthesia) results of less than 5 mm/5 min in at least 1 eye; low tear film breakup time (<5 s); mild superficial punctate keratitis, defined as a corneal punctuate fluorescein staining score of 1 or more in either eye (scale, 0 [none] to 3 [severe]); and symptoms of ocular irritation as assessed by an Ocular Surface Disease Index score [23] of 25 or more (on a scale of 0 through 59). The diagnosis of DED was based on the assessment of the signs and symptoms by 2 observers (K.N. and C.J.). Both examiners analyzed the severity levels of the patients after taking photographs of the anterior segments. The exclusion criteria were as follows: an inflammatory disease not associated with dry eye; ocular trauma or surgical history within the previous year; pregnancy; systemic diseases such as diabetes and hypertension; rheumatologic, hematologic, and respiratory diseases; systemic infection; and any other significant disease. During the study, patients were excluded when they were found to fulfill any of the above exclusion criteria, even though they may not have been aware of fulfilling any of these criteria at the time of enrollment.

Healthy controls were selected from visitors who have regular medical check-ups in the Health Promotion Center of St. Mary’s Hospital in Seoul, Korea. Inclusion criteria were as follows: No subjective ocular symptoms; no abnormal signs of the eye after ophthalmic examiner of the ophthalmologist (K.N.); no ocular trauma or surgical history; no systemic diseases confirmed by physician with their results of medical examinations; and no medication history which can affect the ocular surface physiology.

### Genotyping

Genomic DNA was extracted from peripheral blood samples using the QIAamp DNA blood kit (QIAGEN, Valencia, CA). Polymerase chain reactions (PCRs) were performed with 25 ng of genomic DNA as a template in a mixture of PCR buffer, 2.5 mM MgCl_2_, 200 nM dNTPs, 0.4 pmol of each primer, and 0.75 units of h-Taq polymerase (Solgent, Daejeon, Korea; Table 1). Bilateral polymorphism in the proinflammatory cytokine gene cluster was determined by PCR-restriction fragment length polymorphism (RFLP) analysis and direct sequencing. For screening genetic variations in proinflammatory cytokine genes, the −511 (rs16944) and 31 (rs1143627) positions in the promoter region of *IL1B*, rs1143634 in exon 5 of *IL1B*, rs1800795 in the *IL6* promoter, and Asp358Ala (rs8192284) in *IL6R* were genotyped using PCR, RFLP analysis, and direct sequencing.

**Table 1:** Polymorphic sites of *IL1B, IL6*, and *IL6R* genes.

**Genes**	**position**	**dbSNPs**	**Nucleotide**	**Amino acids**	**RFLP analysis**
*IL1B*	promoter	rs16944	-511C>T		Aval RFLP
*IL1B*	Exon 5	rs1143634	+3954C>T	F105F	TaqI RFLP
*IL6*	promoter	rs1800795	-174G>C		NIaIII RFLP
*IL6R*	Exon 9	rs8192284	c.1073A>C	D358A	HinfI RFLP

### Statistical analysis

The Hardy–Weinberg equilibrium (HWE) was calculated using the GenePop on the Web (GENEPOP; for statistical analysis) web version 4.0 program. To determine statistically significant differences in genotype and allele frequencies between the two groups, we used the χ^2^ test of Fisher’s exact test for the 2x2 contingency table file. The descriptive statistics for observed differences in allele or genotype distribution with the corresponding p value were analyzed using the JavaStat (for odds ratio analysis ) web software in combination with StatXact-8 software (Cytel Inc., Cambridge, MA). The strength of the association was estimated by odds ratio of risk (OR) and 95% confidence intervals (CI). Haplotype frequencies and associations were calculated with Haploview (version 4.0) that ises the expectation maximization (EM) algorithm [23]. Haplotype distribution were evaluated by the permutation test on the basis of 10,000 replications to obtain the empirical significance [24]. Values of p<0.05 were considered statistically significant.

## Results

The tested gene variations are as follows: rs16944 (−511T>C) in the promoter region of *IL1B*, rs1143634 in exon 5 of *IL1B*, rs1800795 (−174G>C) in *IL6*, and rs8192284 (48865A) in *IL6R*. The genotypic and allelic frequencies in *IL1B*, *IL6*, and *IL6R* are listed in Table 1.

**Figure 1:**
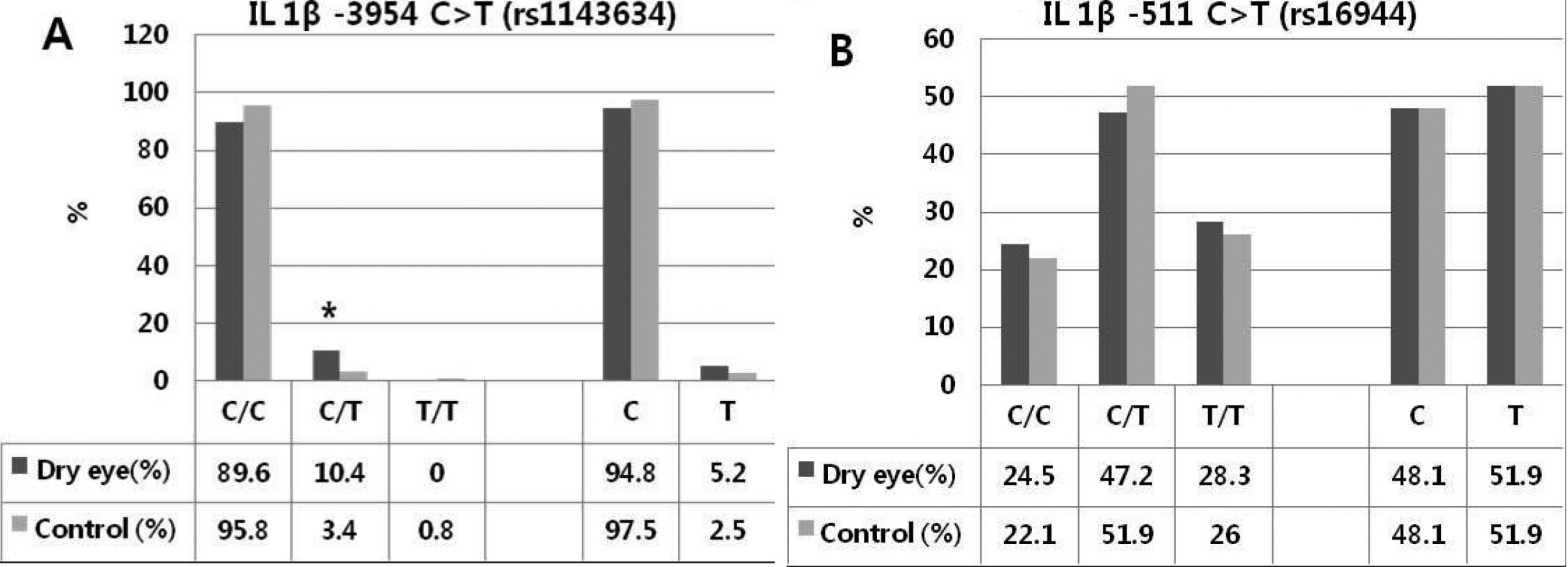
The genotype polymorphism of *IL1β* rs1143634 and *IL1β* rs16944. **A**: The frequencies of *C/*T genotype of *IL1β* rs1143634 in non-Sjogren dry eye patients was increased compared with the control subjects (10.4% versus 3.9%, p=0.043, OR=3.337). **B**: No significant differences were shown in the *IL1β* rs16944 polymorphism.

We found that rs1143634 (F105F) in exon 5 of *IL1B* was significantly different from Hardy–Weinberg equilibrium between DED patients and the controls. The frequency of the CT genotype in DED patients was increased relative to that of the control subjects (10.4% versus 3.9%, p=0.043, OR=3.337; Figure 1). The rs16944 in *ILβ* showed no significant differences in each groups. The genotype distribution of *IL6* (rs1800795) did not show any significant deviation between study and control groups.

**Figure 2:**
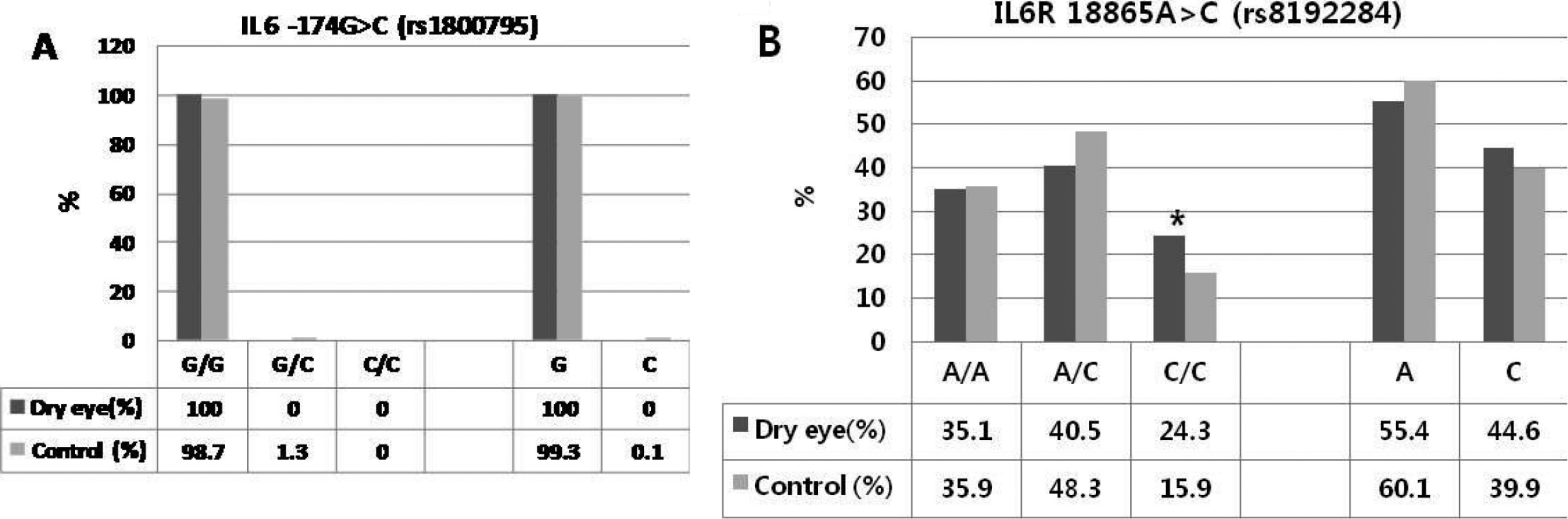
The genotype polymorphism of *IL6* rs1800795 and *IL6R* rs8192284. **A**: No differences in frequencies with rs1800795 in the *IL6* gene. **B**: In the *IL6R* gene, genotypic and allelic distribution of rs8192284 was different between non-Sjogren dry eye patients and controls; *C/*C genotype (p=0.017, OR=2.12) and *C allele (OR=1.26).

For the *IL6R* gene, the genotypic and allelic distribution of rs8192284 was different between the DED patients and the controls: *C/*C genotype (p=0.017, OR=2.12) and *C allele (OR=1.26; Figure 2).

## Discussion

The exact mechanism of DED has not been firmly established. Desiccating environmental stress and changes in tear fluid composition accompanying lacrimal gland dysfunction appear to trigger ocular surface inflammation. Fundamental to the health of the ocular surface is the immune system, which is designed to respond rapidly to environmental and microbial insults. However, sustained inflammation from this response may result in the activation of the autoimmune system to self-antigens localized to the ocular surface [25]. Aqueous-deficient dry eye (ADDE) implies that dry eye is due to a failure of lacrimal galned tear secretion and has 2 major groupings: Sjogren syndrome dry eye and non-Sjogren dry eye [1]. Of the systemic autoimmune diseases that induce lacrimal gland damage, Sjogren syndrome dry eye is likely the best understood one. However, without results from blood tests or other invasive tests, a clinician can have difficulty in identifying whether a patient has Sjogren or non-Sjogren DED since both diseases have similar signs and symptoms. Recently, the new concept has arisen that non-Sjogren DED is a complex, multifactorial autoimmune disorder with a largely unknown pathogenesis. The conditions associated with non-Sjogren DED is primary lacrimal gland deficiency including age-related dry eye, and congenital alacrima; secondary lacrimal gland deficiency including sarcoidosis, lymphoma, and AIDS; obstruction of the lacrimal ducts due to trachoma, erythema multiforme, and chemical or thermal burns; and reflex hyposecretion due to contact lens wear, diabetes, 7th cranial nerve damage, and systemic drugs [1]. The presence of dysfunctional regulatory T cells (Tregs) and the resistance of pathogenic T cells, especially Th17 cells, to Treg suppression was demonstrated in a DED mouse model [26]. Autoimmune diseases, including IMID, are the consequence of an inappropriate immune response directed to self-antigens. The susceptibility of certain individuals to developing an autoimmune disease is associated with multiple genes, as well as other risk factors including environmental triggers. The combined role of genes, environment, and immunity in the development of an autoimmune disease has been the subject of interest for many investigators [27-30].

Most studies have focused on the downstream components of DED pathogenesis, including T cells, cytokines, and chemokines. However, little is known about the genetic factors involved in the susceptibility to the disease. We demonstrated that the genotypic and allelic distribution of rs1143634 (F105F) in *ILβ* and rs8192284 in *IL6R* were significantly different in DED patients compared to healthy controls.

ILβ, a proinflammatory cytokine expressed by activated macrophages and several other types of cells, is thought to play a crucial role in the pathogenesis of autoimmune diseases. IL1βwas initially known as one of the lymphocyte-activating factors (LAFs), owing to its role in the induction of T-cell proliferation and maturation [31,32]. Several reports have predicted the genetic association of *IL1*β with the development of Graves’ ophthalmopathy, ankylosing scleritis, and cystic fibrosis [33-35]. Our results demonstrated that rs1143634 (F105F) in exon 5 of *IL1β* was significantly different between patient and control groups. The frequencies of the C/T genotypes were increased in the DED patients relative to those of the control subjects. It has been reported that the elevated osmolarity achieved by adding sodium chloride to the culture medium of corneal epithelial cells increased the production of IL1β [36]. Using RNA isolated by impression cytology from 15 patients with Sjogren DED, it was shown that ocular surface epitheliopathy, squamous metaplasia, and *IL1β* were highly correlated [37]. The exact mechanism of the C/T genotype of rs1143634 (F105F) in exon 5 of *IL1β* is not confirmed; however, this phenotype may play a role in the upregulation of *IL1β*.

We also observed that the genotypic and allelic distributions of rs8192284 in *IL6R* were different between DED patients and controls: C/C genotype. A single nucleotide polymorphism (rs8192284) has been described to result in an amino acid substitution from aspartic acid to alanine (D358A) [38]. This change has an effect on IL6R shedding, with individuals carrying the minor C allele showing an increased shedding of membrane-bound receptors than the non-C allele carriers [39]. Plasma-soluble IL6R levels have been shown to be positively associated with the number of C alleles in rheumatoid arthritis patients [40]. IL6R is formed by 2 different membrane glycoproteins: IL6Rα an 80-kDa type I protein referred to as the ligand-binding subunit, and IL6Rβ a 130-kDa (gp130) protein referred to as the signal-transducing subunit [41]. IL6 and IL6Rαform a low-affinity complex that must bind with gp130 to form a high-affinity complex that can transduce signals to the cytoplasm. The exact role of IL6 is unclear, and both its pro- and anti-inflammatory properties need to be shown in vivo and in vitro. The previous studies hypothesized that the balance between the pro- and anti-inflammatory effects of IL6 is tilted toward the proinflammatory side under various environmental conditions in DED patients [41,42].

We are the first to report that rs1143634 (F105F) in exon 5 of *IL1β* and rs8192284 of *IL6R* were significantly associated with non-Sjogren DED. Although the pathogenesis of non-Sjogren DED is not clearly understood, our study has revealed that there is a genetic susceptibility to disease occurrence. In addition, the results of the study support the concept of autoimmunity in non-Sjogren DED. Future studies will need to include more subjects and evaluate the corresponding levels of plasma IL1βand IL6R. Moreover, clinical correlations between these allelic variations and disease severity will need to be examined.
